# A child-centred intercultural approach to the socio-educational inclusion of migrant and refugee children

**DOI:** 10.12688/openreseurope.16999.1

**Published:** 2023-12-29

**Authors:** Eva Bajo Marcos, Valeria Fabretti, Ángela Ordóñez-Carabaño, Elena Rodríguez-Ventosa Herrera, Silvia Taviani

**Affiliations:** 1 University Institute of Studies on Migration, Comillas Pontifical University, Madrid, Community of Madrid, 28015, Spain; 2 Save the Children Italia, Roma, 00184, Italy; 3 Psychology, Comillas Pontifical University, Madrid, Community of Madrid, 28049, Spain

**Keywords:** Child-centred approach, migrant children, refugee children, socio-educational inclusion, integration, interculturalism.

## Abstract

The increasing trend of children's migration, whether forced or voluntary, presents a challenge to policies that aim to ensure social cohesion and protect children's rights. Therefore, adopting a child-centred approach to the socio-educational inclusion of migrant and refugee children can aid in the creation of cohesive and inclusive societies.

Inclusive educational environments are collaborative settings that promote participation among children, educators, the community, and other local institutions. Educational communities can play a leading role in implementing public policies that promote social inclusion and intercultural dialogue. Schools play a crucial role in integrating migrant and refugee children. Child-centred practices can be implemented to promote intercultural and linguistic competences, capacity building, and children's agency. This can help to develop a sense of belonging and inclusion for all students. To unlock the potential of inclusive education, reduce inequalities, and achieve more equitable societies, it is essential to integrate a child-centred approach that promotes access to fundamental rights, participation, recognition of cultural diversity, and children's socio-emotional well-being.

This review discusses the challenges of adopting a child-centred approach to education for migrant and refugee children. It proposes inclusive interculturalism as a child-centred approach to address the socio-educational deficits of these children in educational settings in Europe.

## Introduction

Childhood studies and its inclusive Child-Centred Approach (CCA) are emerging as a theoretical and methodological framework that produces meaningful outcomes for children and the wider society ( Qvortrup, 2014). However, it is only recently that migration studies have adopted this perspective to better understand the educational experiences of migrant and refugee children ( Due *et al.*, 2014).

At an international level, the European Education Area (EEA) strategy has placed education at the centre of European social cohesion and development. This has significant implications for the integration processes and educational experiences of migrant and refugee children ( European Union, 2021). The strategy prioritises actions to improve the quality, success, and inclusion of children with a migrant background. It identifies improving access to quality education for these children as a concrete issue and proposes related actions ( European Union, 2021, p. 17). Effective inclusive education and social policies are necessary to ensure the successful integration of children. This includes promoting inclusion in national education systems, providing access to quality education, and creating opportunities for social belonging ( Correa-Velez *et al.*, 2015).

At the community level, the educational experiences of migrant and refugee children are strongly influenced by their educational communities. Local schools play a fundamental role in shaping the integration processes of these children. In terms of social ties and membership, they connect migrant and non-migrant communities. They serve as a bridge between other institutions of the public system and the educational community. Additionally, they provide a safe environment and framework of reality in which migrant and refugee children begin to develop a sense of belonging, supportive relationships, and new cultural identities ( Ahad & Benton, 2018; European Commission/EACEA/Eurydice, 2019). Furthermore, schools have traditionally served as liminal spaces where children from diverse backgrounds can connect, interact, and develop, providing opportunities for their future success ( Clauss-Ehlers *et al.*, 2013). Schools provide a privileged setting for studying the educational experiences of migrant and refugee children and implementing comprehensive interventions to maximize their integration across all dimensions ( Block *et al.*, 2014).

Previous research in this area has identified several challenges at the children's level that affect their education. These challenges arise from their migration experience and the demanding nature of the integration process. The literature emphasises the importance of learning how to assimilate into a new culture, become a member of different communities, adjust to a different educational system, and ultimately develop new identities as full members of a society that is different from one's own ( Due *et al.*, 2016).

In summary, research at the individual, community, and societal levels has been dedicated to understanding the educational experiences of migrant and refugee children. This progress has put the study of migrant and refugee children's education on the map. However, a more comprehensive approach is necessary to link these findings, focusing on the intersecting factors that shape individual experiences and giving voice to the experiences of migrant and refugee children themselves. Despite these knowledge gaps, previous work integrating a CCA into the socio-educational inclusion of migrant and refugee children is lacking ( Due *et al.*, 2016).

This review analyses the socio-educational inclusion of migrant and refugee children from a child-centred perspective. It aims to provide insight into the school lives of children, their needs, and the circumstances that shape them. The subsequent sections present a theoretical discussion of the challenges involved in adopting and implementing a child-centred approach to the education of migrant and refugee children. We propose an inclusive and intercultural approach to address the identified shortcomings and contribute to the theoretical debate. The discussion highlights the potential of inclusive interculturalism as an educational perspective linked to the basic principles of CCA. It presents various dimensions of the socio-educational inclusion of migrant and refugee children in Europe.

## Challenges to child-centred education for migrant and refugee children in Europe

### A child-centred approach to the education of migrant and refugee children

In general, CCA combines a holistic view of children and societies with a rights-based perspective ( Qvortrup, 2014). Children are viewed as biopsychosocial beings at the centre of a nested system of social interactions that enable their rights to be heard and participated in, thus building the necessary capacities to shape their resilience and well-being ( McCarthy & Marks, 2010). Although academic research, psychosocial interventions, and policies focused on children tend to operationalise multiple protective and risk factors at different levels to strengthen their resilience and well-being processes, well-being researchers have emphasised the importance of empowering children as a specific group. This is achieved by promoting their informed control and influence over the factors that affect them ( Solar & Irwin, 2010). Attending school and receiving an education are necessary experiences for children's empowerment. They provide practical opportunities for development, flourishing, and meaningful engagement with their social contexts ( Dryden-Peterson, 2016). 

Schools can adopt various educational approaches based on their philosophy, which can have different implications for their student body. Child-centred education aims to apply the principles of a child-centred approach to education, both conceptually and practically, in schools ( Sedmak *et al.*, 2021). This approach centres on designing educational practices that prioritise the child over the school curriculum. The focus is on learning methods rather than the content to be covered, adapting to the abilities and needs of the children ( Perren *et al.*, 2017; Sutherland, 1995). The literature on child-centred education primarily focuses on early education and care. However, there is a lack of information on how to apply this approach with older children and adolescents ( Perren *et al.*, 2017).

To address this gap, some authors have attempted to incorporate more elements of the child-centred approach in educational settings, including the notions of agency and participation. These perspectives view children as active participants and the most reliable source of information on all matters that affect them ( Mayeza, 2017). The basic principle is that considering their perspectives and allowing them to generate and exchange their own interpretations will enhance their self-esteem and overall welfare. Moreover, it will facilitate communication with their peers and, collectively, foster the development of problem-solving abilities ( Gornik & Sedmak, 2021).

Although many consider this approach positive for children's overall development and well-being, it is primarily a theoretical perspective. It serves as a general guiding principle that requires adaptation for practical implementation. The evidence and practical guidelines for its implementation are still insufficiently developed ( Gornik & Sedmak, 2021). Migrant and refugee children face several barriers to inclusion in the classroom, hindering their ability to benefit from a child-centred approach. When a newly arrived migrant child begins school in a host country, they must overcome challenges such as learning the host language, making new friends, and dealing with acculturative stress ( Fernández García *et al.*, 2019; Martin *et al.*, 2019). In modern schools, adopting a CCA can help to address the differential level of competence development and environmental capacities among diverse student bodies.

Migrant and refugee children may experience discrimination due to various factors such as geographical, identity, and categorical boundaries, as well as other social factors ( Kern *et al.*, 2020). Previous studies that applied an intersectional perspective have demonstrated the complex ways in which age, gender, ethnicity, religion, disability, or national and economic status interact, generating intra- and inter-group dynamics of exclusion. For example, in a specific context, female migrant and refugee children may have fewer opportunities for social interactions than male migrant and refugee children of the same age and ethnicity ( Bastianelli, 2015). A child-centred approach to educating migrant and refugee children must consider the variability in their educational experiences and focus on promoting agency and participation in the school context. If done correctly, this approach can empower migrant and refugee children by creating an environment that is open to listening to their voices and facilitating the development of individual resilience skills ( Messiou *et al.*, 2022). To the best of our knowledge, there are currently no child-centred educational interventions that specifically target migrant and refugee children and provide practical guidelines in this regard.

All children have the right to be heard and to participate in the matters affecting their well-being and future trajectories in life ( United Nations, 1989). However, barriers to inclusive education can prevent migrant and refugee children from influencing their context or expressing themselves, leading to their educational experiences being overlooked ( Power *et al.*, 2019). The well-being and agency of migrant and refugee children can only be achieved if they are given the opportunity to fully participate alongside their non-migrant peers. This requires addressing their personal needs as children first, and then acknowledging their status as migrants and the potential educational disadvantages they may face due to their background ( Kayaalp, 2014). The failure to listen to the voices of migrant and refugee children can result in their expressed needs being overlooked, perpetuating disadvantage and inequality ( Messiou *et al.*, 2022).

Therefore, policy, research, and practice should prioritize capacity building to enhance individuals' personal resources and skills to cope with life challenges in a resilient and agentic manner. Additionally, it is crucial to create responsive environments that offer opportunities, resources, and pathways for both individual and social development ( Ayala-Nunes *et al.*, 2018).

### Barriers to education for migrant and refugee children in Europe

The ecological theory proposes that a child's development is influenced by a nested structure of social systems that extend from the proximal and most significant settings and figures of interaction with the child (micro and meso levels) to the broader and more distant socio-cultural context (exo and macro levels) ( Bronfenbrenner & Morris, 2007). In parallel, the rights-based approach considers the empowerment of children through their participation and influence in the social context to which they belong ( Camfield *et al.*, 2009). Reflecting on the education of migrant and refugee children from a child-centered approach links both fields. It emphasizes the importance of allowing children to represent their own needs. By considering children as capable of defining their interests and providing them with opportunities to be heard across different social levels, their social and personal empowerment can be enhanced ( Sedmak *et al.*, 2021). The agentic view of childhood, which is present in children's legislation and social sciences, should also be adopted in education to enhance the inclusion, empowerment, healthy development, and quality of life of migrant and refugee children ( Hannah, 2007). However, achieving this goal is not without challenges.

Structural barriers at the macro level have the broadest range of influence on all the embedded systems of interaction that affect children, thereby shaping their educational experiences. The implementation of children's rights is necessarily linked to understanding the diverse social conditions of childhood. It is important to make these underpinnings explicit to make visible the realities of migrant and refugee children in a reliable way ( Mayall, 2002). When discussing the rights of migrant and refugee children, it is crucial to consider their inclusion as full members of society, with the same rights as non-migrant children. The intersection of their social identities as children and migrants can hinder their ability to access educational opportunities and experiences equitably ( Ulloa-Cortés, 2021). Although European policymaking places great emphasis on the integration of migrants and highlights equality and intercultural dialogue as the cornerstone principles for embracing the increasing diversity of European societies ( Council of Europe, 2008), migrant and refugee children still experience educational inequality ( European Commission/EACEA/Eurydice, 2019). Successful inclusion requires effective political leadership that encompasses official educational standards and social inclusion agendas. To foster inclusion, it is necessary to address policies as well as practices ( Ainscow, 2016).

First, migrant and refugee children are especially vulnerable as their basic needs and personal development depend on the degree of recognition of their legal status and social rights by the host country. Migrant and refugee children are especially vulnerable as their basic needs and personal development depend on the degree of recognition of their legal status and social rights by the host country. Migrant and refugee children are especially vulnerable as their basic needs and personal development depend on the degree of recognition of their legal status and social rights by the host country. This recognition process is often influenced by the political will of those in power ( García Cívico, 2010). Rights and citizenship form the foundation for establishing expectations and obligations for the inclusion process ( Ager & Strang, 2004). Migrant children who access educational systems in vulnerable positions due to legal status, such as asylum-seekers, migrants in an irregular situation, and unaccompanied minors, are at a significantly higher risk of early school leaving and educational underachievement ( European Commission/EACEA/Eurydice *et al.*, 2019).

Secondly, there are also barriers at the meso level that may have a more limited range of influence. However, as this level represents children's interpersonal relationships and communities, they hold the potential for a more profound effect on children. Therefore, emphasis has been placed on children's participation in their educational community and schools as sources of psychosocial support that provide a safe context for development ( Vilela *et al.*, 2018).

Mainstream schools are formal educational settings that receive resources to provide all children with equitable quality education and promote their future learning opportunities. Migrant and refugee children may experience psychological distress due to previous traumatic experiences, difficulties adjusting to a new language and culture, family separation, and discrimination ( Bronstein & Montgomery, 2011; Fazel *et al.*, 2012). Particularly, refugee children are at a greater risk of developing emotional and behavioural difficulties due to the multiple losses they experience ( Beiser & Hou, 2016).

Schools can provide a safe environment to address potential problems by offering *psychosocial support services*, such as counselling or therapy. Providing these services within the school can be particularly beneficial as families and children may be hesitant to attend external mental health services, but may feel more comfortable accessing them at school. Furthermore, incorporating social and emotional skills into school curricula can enhance the resilience of migrant and refugee children and positively impact their well-being ( Fazel & Betancourt, 2018; Heidi *et al.*, 2011).

*Effective leaderships* crucial in schools. Inclusive education demands culturally responsive leadership that is dedicated to achieving equity, inclusion, and equal opportunities for all students while embracing the local school culture's agendas ( Khalifa *et al.*, 2016). This can only be achieved through the commitment of school principals and management teams, collaborative teamwork among school staff, and their appropriate training in cultural responsiveness ( Shaw, 2017). These factors entail a shared framework among school staff that maintains a positive attitude towards students and their learning abilities ( Topping & Maloney, 2005). Adequate training of the teaching staff is critical for effective inclusive education, as teachers should possess sufficient knowledge about learning difficulties and skills to develop specific instructional methods ( Forlin, 2010).

Besides, intensive *learning support* should be provided by schools to ease the cognitive and emotional challenges faced by newly arrived migrant and refugee children ( Sinkkonen & Kyttälä, 2014; Taylor & Sidhu, 2012). Preparatory classes are particularly important at the secondary level, where students face greater difficulties in acquiring a new language and the curriculum demands language proficiency ( Crul *et al.*, 2019). However, extended preparatory classes could potentially hinder integration by separating migrant and refugee children from their native peers or delay other academic learning by focusing solely on language acquisition ( Nilsson & Bunar, 2016).

European countries have committed to implementing inclusive educational approaches. However, *physical and social segregation* often occurs due to housing segregation, which, in turn, leads to school segregation ( Agirdag *et al.*, 2011; Lindgren, 2010; Slee, 2012). Migrant students are particularly affected by the concentration of socio-economically disadvantaged neighbourhoods and schools with lower academic standards, which negatively impacts their educational achievement. Empirical studies have shown that children from ethnic and lower socio-economic minorities are disproportionately represented in special schools, where they often experience academic underachievement ( Kavale, 2007; Nusche, 2009). The findings suggest that better academic, social, and personal outcomes can be achieved through inclusion in mainstream schools with additional resources. However, better results are mainly determined by the quality of the provided resources ( Shaw, 2017).

For this reason, non-formal educational settings are particularly relevant for migrant and refugee children. These spaces provide alternative educational opportunities and offer more nuanced attention to their educational needs. They also create effective bridges and bonds with the community, which eases their social inclusion ( Wiktorin, 2017). In this respect, the Sirius report “Role of non-formal education in migrant and refugee children inclusion: links with schools” emphasises the importance of collaboration between formal and non-formal educational settings in promoting the overall development of children. This collaboration can help foster inclusion, well-being, and academic achievement ( Lipnickienė *et al.*, 2018). In fact, This report demonstrates that non-formal education can facilitate inclusion by bridging with formal education, combating segregation, providing academic and emotional support, offering linguistic support, overcoming trauma, building resilience, promoting intercultural competencies, and preventing radicalisation and violence. Despite the benefits, non-formal education is often less visible, less recognised, and harder to validate ( Souto-Otero & Villalba-Garcia, 2015).

In conclusion, negative attitudes towards migrants and refugees can hinder their ability to have an optimal educational experience. This can have an impact on both macro and meso levels, ultimately affecting the migrant children themselves. Discrimination experiences can significantly alter the attitudes and orientations of migrants and refugees towards mainstream society, which can reduce their motivation to integrate into the host culture ( Lutterbach & Beelmann, 2021). Additionally, bullying or discriminatory treatment by peers, whether from the host society or their own ethnic group, can have devastating consequences on the relationship between migrant and refugee children and their peer group ( Kang, 2010).

## Inclusive interculturalism: addressing the socio-educational shortcomings of migrant and refugee children in Europe

As explained in the previous section, children's educational experiences are complex and involve multiple parties and key figures that influence how children engage with, feel about, and develop their learning trajectories ( Ballaschk & Anders, 2020). The barriers and gaps to socio-educational inclusion discussed in this text represent significant obstacles to the learning and participation of migrant and refugee children. These obstacles are based on the different identities and social labels that intersect with them. To solve these problems, it is necessary to adopt an inclusive and intercultural approach to education that prioritises the social and personal needs of children. This is in line with the Incheon Framework and strategies for sustainable development and education ( UNESCO, 2016).

The strategy for future actions in the development of educational inclusion and equity stresses the need to ensure access to education for all students, particularly those in vulnerable situations, and to strengthen the quality of learning and skills ( UNESCO, 2016). Therefore, in order to address the educational challenges faced by migrant and refugee children, it is necessary to remove barriers to education by effectively implementing inclusive education. Additionally, it is important to be aware of the inequalities that arise from coming from a different socio-cultural context, which can be achieved by adopting an intercultural model for educational inclusion ( IOM, 2019).

Hereafter, we will provide an in-depth discussion of these concepts to offer insight into a child-centred definition of socio-educational inclusion for migrant and refugee children that embodies inclusive interculturalism in education.

### An analysis of the contribution of intercultural education

Interculturalism is a crucial model for inclusive education that concentrates on developing cultural competences to promote peacebuilding and understanding among students from diverse backgrounds. This approach goes beyond passive coexistence and aims to establish a sustainable way of living together in multicultural societies through mutual understanding, respect, and dialogue between different cultural groups ( UNESCO, 2010). The UNESCO’s landmark document *Guidelines on Intercultural Education* ( UNESCO, 2006) state that applying an intercultural approach to educational contexts requires respecting the cultural background of all students and providing them with culturally appropriate and responsive quality education. To achieve this, students must be provided with cultural knowledge and taught to develop positive cultural attitudes and skills that enable them to intentionally recognise identities as multiple, hybrid, and complex. This will help them avoid categorisations and develop solidarity and respect towards diverse groups of people ( Belmonte, 2017).

Removing barriers to the education of migrant and refugee children requires fostering intercultural dialogue within school cultures. The increasingly diverse student bodies in educational settings are a current concern that impacts the school climate and the psychosocial well-being of both migrant and non-migrant background students ( OSCE Office for Democratic Institutions and Human Rights (ODIHR), 2018). ntercultural dialogue in this context facilitates the exchange of perspectives and knowledge, with the aim of achieving mutual understanding and respect ( Council of Europe, 2008). Therefore, intercultural dialogue is a crucial tool for promoting more inclusive educational environments for migrant and refugee children.

Introducing children to core values, attitudes, and skills and encouraging their adoption beyond school settings is fundamental for developing intercultural competence. This vision motivates children to embrace their differences and similarities with others, creating a solid foundation for respect and the creation of more inclusive and cohesive societies ( Essomba, 2014). In this sense, Intercultural education is child-centred, highlighting the role of children as active participants and shapers of their own realities. It emphasises that children are reliable experts and acknowledges their influence not only on their own lives but also on the lives of those around them and ultimately on society ( Aguado-Odina & Sleeter, 2021).

Intercultural education is a form of educational inclusion, as stated in the specialised literature, and is considered one of the best ( Arroyo, 2013). The guiding principle of inclusive education is equity. Attention is paid to the particular educational needs of students, seeking to unveil how they are socially conditioned. From this perspective, intentional efforts are made to reverse inequality and enhance students' competence to develop and function across diverse sociocultural environments. In this context, equality refers to the availability of opportunities to make informed choices and access social, economic, and educational resources. The international consensus is to shift the focus from diverse students to local mainstream schools ( Ainscow, 2020). From this perspective, inclusion is about creating school cultures that encourage the reassessment of learning conditions offered to students. This includes identifying specific barriers to participation and learning, fostering collaboration among all members of the educational community, sharing practices and ideas, promoting innovation, building upon previous knowledge and practice, and making use of available resources for learning support ( Ainscow & Sandill, 2010).

Therefore, it is important to recognize the systems that directly impact children's adaptation in order to develop intercultural competence. Migrant and refugee families are rooted in cultural and social contexts that differ significantly from those of native families, resulting in the formation of distinct identities. Inclusive intercultural education involves adopting a pluralistic perspective that acknowledges the diversity of experiences and benefits all students ( Moser *et al.*, 2017).

### A discussion of socio-educational inclusion for migrant and refugee children

The previous sections have shown that a multidimensional perspective is necessary for accurately understanding the socio-educational inclusion of migrant and refugee children. Based on key literature and research, five interrelated dimensions that are crucial for the successful inclusion of migrant children in society have been identified: legal status, linguistic competences, psycho-social wellbeing and health, social relations, and educational achievements ( Serrano Sanguilinda *et al.*, 2019). Considering these dimensions can help develop a more accurate child-centred view of the socio-educational inclusion of migrant children, addressing gaps that have been identified. Additionally, taking into account the children's perspective when examining these dimensions may advance migration studies, which have traditionally focused on adults. 

Attention to migrant children's *access to rights* reveals that their basic needs, personal development, and social inclusion depend primarily on the degree of recognition given to their migratory status by the host society and the set of rights, expectations, and obligations attributed to it. This is also dependent on the prevailing idea of citizenship ( Ager & Strang, 2004; Bauböck, 2003). From a child-centred perspective, this dimension of integration relies on the fundamental supporting principle that international and EU legislation prioritises the status of children over that of migrants in defining and attributing a set of rights. These rights include those enshrined in the United Nations Convention on the Rights of the Child. This set includes the right to access education, healthcare, and other fundamental rights. State authorities must always prioritize the best interest of the child in any administrative decision ( UNHCR, 2008). However, to understand this dimension from a child's perspective, it is important to consider the various situations and ways in which migrant children may be affected by external variables, such as national legislation on migration and citizenship, as well as personal variables, such as family migration history, origin, and age, which may impact their access to rights. This is a significant issue, particularly in the case of unaccompanied minors. Their age range and assessments often categorise them as adults or nearly-adults, which may hinder their integration paths.

*Linguistic competence*, specifically the acquisition of the national language of the host society, is a crucial prerequisite for integration. As Heckmann (2008) has demonstrated, factors such as a young age, a solid foundation in a mother tongue, a certain degree of family cultural capital, and the provision of language classes by schools are essential in achieving this goal. The CCA recommends emphasising the significance of linguistic competence in ensuring children's autonomy, voice (i.e. the expression of their views and needs), and social participation. Additionally, it is important to incorporate children's perspectives when addressing language issues in both formal and informal educational settings. Educators should recognise and value children's competences in their first language, and promote plurilingualism as a tool for educational inclusion and intercultural education. UNESCO has advocated for home language teaching in pre-primary and primary education since 1953. Scientific research has consistently highlighted the positive effects of such teaching on students' social, cognitive, and linguistic development ( European Commission/EACEA/Eurydice, 2019).

The primary outcomes of integration are *health and psychosocial well-being*. Integration can be interpreted as a process of social inclusion that helps individuals meet their basic needs ( Harttgen & Klasen, 2009). The relationship between integration and well-being is particularly evident when child-centred perspectives are adopted instead of adult-centric approaches. The assessment of migrant children's integration, particularly in connection with the social dimension, highlights the importance of aspects such as children's happiness, self-esteem, and sense of belonging ( Bajo Marcos *et al.*, 2022). Besides, the literature widely studies the areas where well-being and group identity overlap. Positive feelings of belonging may help counteract the negative consequences of outside threats ( Liu & Zhao, 2016) and may play a key role in maintaining psychological health ( Eccles *et al.*, 2006). The CCA aims to expand on this concept by analysing the agency and resilience of migrant children ( Fonagy *et al.*, 1994) in host societies. It is important to consider age, as adolescence is a particularly vulnerable stage in emotional development, during which migrant children may experience feelings of vulnerability, exclusion, and lack of confidence ( Perez, 2016). Child-accessible psycho-social support measures, such as counselling or therapeutic services at schools, can enable children to take an active role in caring for their own mental health. This is particularly important in cases where accessing health services for children and families is critical. Teaching social and emotional competences, creating inclusive and integrative climates at school, and promoting social connectedness with both peers and teachers can improve migrant students' resilience and positively impact their academic outcomes ( European Commission/EACEA/Eurydice, 2019).

*Social relations*, including bonds, bridges, and links, form the structural foundation of social capital. They serve as pathways for the transmission of values, attitudes, and behaviours ( McLeod & Lively, 2003). ocial bonds with peers and teachers, as well as links with institutions, are particularly important outcomes of migrant children's integration. However, factors such as low family status can affect trust in institutions and the size of the school network among migrant students ( Santagati, 2015). Non-inclusive school curricula and climates ( Agirdag *et al.*, 2011), and the spread of societal negative representations and prejudices towards migration can also be barriers to integration ( Banks, 1995). The CCA framework highlights the role of children as competent social actors who can participate in decision-making regarding their social lives ( James & Prout, 1997). Additionally, the focus on agency leads to a non-essentialist view of children's cultural identities as always hybrid and negotiated within continuous interactions with groups ( Baraldi, 2021). In a CCA, it is crucial to ensure that children who are at high risk of exclusion actively participate in their daily interactions. This requires recognising diversity as a positive value, particularly in educational settings, and actively contributing to creating pluralist cultures where all children, regardless of their origin, culture, ethnicity, beliefs, gender, etc., are included and can participate in the community.

Finally, children with migrant backgrounds face significant challenges and difficulties, and they systematically achieve lower *academic results* than native children without an immigrant background ( OECD, 2016). Academic success is dependent on various situational factors, including psychological well-being, emotional development, language acquisition, and relevant social factors. Family cultural capital, in particular, plays a crucial role in shaping motivation and aspirations. The influence of a migrant background on students' learning opportunities appears to be more dependent on socio-economic status than the migration process itself ( European Commission/EACEA/Eurydice, 2019). Successful socio-economic integration of parents reduces the risk of drop-out and educational segregation in vocational schools. The CCA highlights the importance of involving parents in both formal and non-formal educational institutions, as well as encouraging children to actively participate in educational communities. 

The outcomes mentioned are complex and multidimensional. Therefore, it is necessary to consider different levels of analysis for each of them. Furthermore, their various components and determinants are interrelated. For example, well-being outcomes have a significant impact on social relations, and both, in turn, affect academic achievement. Finally, the processes that mediate inclusion outcomes, through the presence of facilitators and barriers, take place and can be observed in different settings within the social system. The CCA emphasises the need for a rigorous consideration and in-depth empirical exploration of the first-hand experiences of migrant children and their understanding of the integration process.

## Conclusions

This review presents a theoretical discussion of the open debates surrounding the specific challenges experienced by migrant and refugee children in education. It explores how their socio-educational inclusion may help to alleviate these shortcomings. The first section reviews child-centred education and the limited development of conceptual work and guiding methodologies for migrant and refugee students. Without age-appropriate and culturally sensitive theoretical frameworks that acknowledge the social conditions and subjectivities of migrant and refugee children, empirical research aimed at evidence-based educational practices is flawed. In addition, as migrants and refugees, these children experience barriers to their inclusion in education, preventing them from fully engaging and benefiting from it. In this article, we examine the barriers that affect migrant and refugee children's access to child-centred education. We explore how macro and meso level factors have both proximal and distant effects on this issue.

Reflecting on these challenges, we propose inclusive interculturalism as a child-centred approach to address the socio-educational shortcomings of migrant and refugee children in educational settings in Europe. This text presents the positive contribution of intercultural education to children's development. It highlights the growth of cultural awareness and intercultural skills, as well as the promotion of children's agency through the underlying values of intercultural education. The text is clear, concise, and uses objective language with a formal register. The grammar, spelling, and punctuation are correct. No changes in content were made. In conclusion, we examine the various aspects of socio-educational inclusion for migrant and refugee children. We connect the narratives of migration and childhood studies to identify new research areas that aim to reduce educational inequalities experienced by these children. This theoretical discussion can serve as a foundation for designing intervention models for child-centred education of migrant and refugee children in future works.

## Ethics and consent

Ethical approval and consent were not required.

## Data Availability

No data are associated with this article.
