# A child-centred intercultural approach to the socio-educational inclusion of migrant and refugee children

**DOI:** 10.12688/openreseurope.16999.2

**Published:** 2024-10-17

**Authors:** Eva Bajo Marcos, Valeria Fabretti, Ángela Ordóñez-Carabaño, Elena Rodríguez-Ventosa Herrera, Silvia Taviani

**Affiliations:** 1 University Institute of Studies on Migration, Comillas Pontifical University, Madrid, Community of Madrid, 28015, Spain; 2 Save the Children Italia, Roma, 00184, Italy; 3 Psychology, Comillas Pontifical University, Madrid, Community of Madrid, 28049, Spain

**Keywords:** Child-centred approach, migrant children, refugee children, socio-educational inclusion, integration, interculturalism.

## Abstract

The increasing trend of child migration, whether forced or voluntary, poses a challenge to policies aimed at ensuring social cohesion and protecting children's rights. Inclusive educational environments play a crucial role in the integration of migrant and refugee children by promoting intercultural dialogue and participation.

This study aims to explore the potential of a child-centred approach to the socio-educational inclusion of migrant and refugee children in Europe, proposing a framework of inclusive interculturalism to address the specific challenges these children face in educational settings.

A narrative review was conducted, examining a wide range of peer-reviewed sources to synthesise current knowledge on child-centred education and its applicability to migrant and refugee children.

The review identifies key challenges in adopting a child-centred approach, including socio-cultural, linguistic and psychological barriers that hinder migrant and refugee children's full participation in education. It highlights the importance of promoting agency, cultural competence and emotional well-being through inclusive and intercultural educational practices.

Implementing a child-centred, inclusive intercultural approach can promote social cohesion and reduce inequalities by ensuring that all children, regardless of their background, have access to quality education and opportunities for personal development. However, more empirical research is needed to translate these theoretical frameworks into practical interventions.

## Introduction

While migrant ^1^ and refugee ^2^ children increasingly populate schools across Europe, current educational policies and practices often fail to address their unique socio-educational needs adequately, leading to lower educational attainment ( Eurostat, 2024). Childhood studies and its inclusive Child-Centred Approach (CCA) are emerging as a theoretical and methodological framework producing meaningful outcomes for children and the wider society ( Qvortrup, 2014). However, it is only recently that migration studies have adopted this perspective to better understand the educational experiences of migrant and refugee children ( Due *et al.*, 2014).

In this context, and at a macro level, the European Education Area (EEA) international strategy has placed education at the centre of European social cohesion and development, which has significant implications for the integration processes and educational experiences of migrant and refugee children ( European Union, 2021). In this sense, the strategy identifies the need to improve access to quality education for children with a migrant background as a concrete and current issue and proposes prioritising actions to boost quality education to enhance students’ success and inclusion ^3^ ( European Union, 2021, p. 17). Social and educational policies striving for inclusion are paramount to ensure that children reach their full potential, and this includes promoting inclusion in national education systems, providing access to quality education, and creating opportunities for social belonging ( Correa-Velez *et al.*, 2015).

At the meso level, the educational experiences of migrant and refugee children are strongly influenced by their educational communities. In this regard, local schools play a fundamental role in shaping these children's integration processes. In terms of social ties and membership, they connect migrant and non-migrant communities and serve as a bridge between other institutions of the public system and the educational community. Additionally, they provide a safe environment and framework of reality in which migrant and refugee children begin to develop a sense of belonging, supportive relationships, and new cultural identities ( Ahad & Benton, 2018; European Commission/EACEA/Eurydice, 2019). Furthermore, schools have traditionally served as liminal spaces where children from diverse backgrounds can connect, interact, and develop, providing opportunities for their future success ( Clauss-Ehlers *et al.*, 2013). Accordingly, schools are a privileged setting, on the one hand, to study how policy and practice influence the educational experiences of migrant and refugee children and, on the other hand, to implement comprehensive interventions to maximise their integration across all dimensions ( Block *et al.*, 2014).

Lastly, previous research in this area has identified several challenges at the children's level that affect their educational outcomes, arising from their migration experience and the demanding nature of the integration process. In this regard, the literature emphasises the importance of learning how to adjust to a new culture, become a member of different communities, adapt to a different educational system, and ultimately develop new identities as full members of a society that is different from one's own ( Due *et al.*, 2016).

In summary, extensive individual, community, and societal research has been dedicated to understanding the educational experiences of migrant and refugee children. This progress has put the study of migrant and refugee children's education on the map. However, a more comprehensive approach is necessary to link these findings, focusing on the intersecting factors that shape individual experiences and giving voice to the experiences of migrant and refugee children themselves. Despite these knowledge gaps, previous work integrating a CCA into the socio-educational inclusion of migrant and refugee children is lacking ( Due *et al.*, 2016). This paper seeks to illuminate these gaps by (1) exploring the challenges of adopting a child-centred approach to education for migrant and refugee children in Europe and (2) proposing a framework for inclusive interculturalism that addresses these challenges. To do so, we conduct a narrative review that would allow us to identify current challenges and discuss a conceptual framework to address these shortcomings. We propose an inclusive and intercultural approach and contribute to the theoretical debate highlighting the potential of inclusive interculturalism as an educational perspective linked to the basic principles of CCA. The conclusions reached are expected to provide insight into children's school lives, their needs, and the circumstances that shape them.

## Methodology

To explore and contextualise the challenges of adopting a CCA to education for migrant and refugee children, we have implemented a narrative review design. Adhering to best practice recommendations, this design is optimal for bridging disciplinary knowledge by offering an in-depth qualitative synthesis from diverse methodological approaches and evidence ( Siddaway *et al.*, 2019). In this manner, we searched 27 indexed and peer-reviewed sources to present a broad perspective on this subject, including Annual Reviews, ERIC, India Database, JSTOR Arts & Sciences I Collection, JSTOR Arts & Sciences II Collection, JSTOR Business I Collection, Latin American & Iberian Database, Linguistics Database, Middle East & Africa Database, Political Science Database, Project Muse, ProQuest Central (new), ProQuest Central, ProQuest Research Library, ProQuest Social Science Journals, PsycARTICLES, Psychology Database, PsycInfo, SAGE Humanities and Social Science (HSS) Package, Scopus, Sociology Database, SpringerLink, Turkey Database, UK and Ireland Database, Web of Science, Wiley Online Library and WorldCat.org. The types of publications spanned from journal articles, books, and book chapters to official reports and doctoral thesis ( Noble & Smith, 2018). In line with this review design, eligibility was established based on the relevance of sources to address the topics of child-centred education and the inclusion of migrant and refugee children. Empirical and theoretical works were considered without temporal constraints.

## Challenges to child-centred education for migrant and refugee children in Europe

### A child-centred approach to the education of migrant and refugee children

CCA combines a holistic view of children and societies with a rights-based perspective ( Qvortrup, 2014). In this perspective, children are viewed as biopsychosocial beings at the centre of a nested system of social interactions that enable their rights to be heard participate, thus building the necessary capacities to shape their resilience and well-being ( McCarthy & Marks, 2010). Academic research, psychosocial interventions, and policies focused on children tend to operationalise multiple protective and risk factors at different levels to strengthen their resilience and well-being processes. On this matter, well-being researchers have emphasised the importance of empowering children as a specific group by promoting their informed control and influence over the factors that affect them ( Solar & Irwin, 2010).

Schools can adopt various educational approaches based on their philosophy, which can have different implications for their student body. There is a consensus that attending school and receiving an education are necessary experiences for children's empowerment, as they provide practical opportunities for development, flourishing, and meaningful engagement with their social contexts ( Dryden-Peterson, 2016). In this spirit, child-centred education aims to apply the principles of a child-centred approach to education, both conceptually and practically, in schools ( Sedmak *et al.*, 2021). This approach centres on designing educational practices that prioritise the child’s needs and incorporate an inclusive curriculum that recognises each child’s rights and enables their participation. The focus is on learning methods rather than the content to be covered, adapting to the abilities and needs of the children ( Perren *et al.*, 2017; Sutherland, 1995).

However, the literature on child-centred education primarily focuses on early education and care, lacking information on how to apply this approach with older children and adolescents ( Perren *et al.*, 2017). To address this gap, some authors have attempted to incorporate more elements of the child-centred approach in educational settings, including the notions of agency and participation. These perspectives view children as active participants and the most reliable source of information on all matters that affect them ( Mayeza, 2017). The basic principle is that considering their perspectives and allowing them to generate and exchange their own interpretations will enhance their educational outcomes, self-esteem, and overall welfare. Moreover, it will facilitate communication with their peers and, collectively, foster the development of problem-solving abilities ( Gornik & Sedmak, 2021). This approach is frequently regarded as beneficial for children's holistic development and well-being. Furthermore, it is supported by a substantial body of literature that provides both theoretical and practical insights. Clark's work, including her Mosaic Approach ( Clark, 2004) and her advocacy for slow pedagogies in early childhood education ( Clark, 2022), provides robust frameworks for effectively implementing child-centred methods. Similarly, Nunn's participatory arts-based studies with resettled refugee young people provide effective strategies for fostering inclusion and belonging ( Nunn, 2018; Nunn, 2022). Recent studies, such as the INNO4DIV Project on the educational needs of teachers for inclusive education in a context of diversity ( Shuali Trachtenberg *et al.*, 2020), provide further evidence of the practical applicability of child-centred approaches. These works provide evidence and practical guidelines that address some of the previously identified gaps ( Gornik & Sedmak, 2021).

Still, migrant and refugee children encounter several obstacles to inclusion in the classroom, which impede their capacity to benefit from a child-centred approach. Upon arrival in a host country, newly arrived migrant children must overcome a number of challenges, including learning the host language, forming new friendships and dealing with the stress associated with cultural adaptation ( Fernández García *et al.*, 2019; Martin *et al.*, 2019). In contemporary educational institutions, implementing a child-centred approach (CCA) can assist in addressing the disparate levels of competence development and environmental capacities among diverse student populations. Additionally, migrant and refugee children may experience discrimination due to various factors such as geographical, identity, and categorical boundaries, as well as other social factors ( Kern *et al.*, 2020). Previous studies that applied an intersectional perspective have demonstrated the complex ways in which age, gender, ethnicity, religion, disability, or national and economic status interact, generating intra- and inter-group dynamics of exclusion. For example, in a specific context, female migrant and refugee children may have fewer opportunities for social interactions than male migrant and refugee children of the same age and ethnicity ( Bastianelli, 2015). A child-centred approach to educating migrant and refugee children must consider the variability in their educational experiences and focus on promoting agency and participation in the school context. If done correctly, this approach can empower migrant and refugee children by creating an environment that is open to listening to their voices and facilitating the development of individual resilience skills ( Messiou *et al.*, 2022). To the best of our knowledge, there are currently no child-centred educational interventions that specifically target migrant and refugee children and provide practical guidelines in this regard.

All children have the right to be heard and to participate in the matters affecting their well-being and future trajectories in life ( United Nations, 1989). However, barriers to inclusive education can prevent migrant and refugee children from influencing their context or expressing themselves, leading to their educational experiences being overlooked ( Power *et al.*, 2019). The well-being and agency of migrant and refugee children can only be achieved if they are given the opportunity to participate alongside their non-migrant peers fully. This requires addressing their personal needs as children first and then acknowledging their status as migrants and the potential educational disadvantages they may face due to their background ( Kayaalp, 2014). The failure to listen to the voices of migrant and refugee children can result in their expressed needs being overlooked, perpetuating disadvantage and inequality ( Messiou *et al.*, 2022). Therefore, policy, research, and practice should prioritise capacity building with a systemic spirit in which the different actors and environments where the child is embedded collaborate to provide equitable participation opportunities that enable children to assume an agentic role. This strategy will enhance the individuals' resources and skills, enabling them to cope with life challenges in a resilient and agentic manner. It is also crucial to create responsive environments that offer opportunities, resources, and pathways for individual and social development ( Ayala-Nunes *et al.*, 2018).

### Barriers to education for migrant and refugee children in Europe

The ecological theory proposes that a child's development is influenced by a nested structure of social systems that extend from the proximal and most significant settings and figures of interaction with the child (micro and meso levels) to the broader and more distant socio-cultural context (exo and macro levels) ( Bronfenbrenner & Morris, 2007). In parallel, the rights-based approach considers the empowerment of children through their participation and influence in the social context to which they belong ( Camfield *et al.*, 2009). Reflecting on the education of migrant and refugee children from a child-centred approach links both fields. It emphasises the importance of allowing children to represent their own needs. By considering children as capable of defining their interests and providing them with opportunities to be heard across different social levels, their social and personal empowerment can be enhanced ( Sedmak *et al.*, 2021). The agentic view of childhood, which is present in children's legislation and social sciences, should also be adopted in education to enhance the inclusion, empowerment, healthy development, and quality of life of migrant and refugee children ( Hannah, 2007). However, achieving this goal is not without challenges.

Successful inclusion requires effective political leadership encompassing official educational standards and social inclusion agendas. To foster inclusion, it is necessary to address policies as well as practices ( Ainscow, 2016). In these terms, successful inclusion is considered as the diverse ways to respond to diversity removing barriers to access, participation and achievement and allowing all individuals to realise their full potential and rights ( Ainscow, 2020). It involves capacity-building, understood in this context as developing environments where the resources, processes and exchanges across systemic levels are oriented towards opportunity creation ( Kendall *et al.*, 2012). This commitment to inclusion is characterised by actions that ensure equal opportunities for all, fostering a sense of belonging, and regularly assessing and adjusting practices to meet the evolving needs of a diverse population.

Structural barriers at the macro level have the broadest range of influence on all the embedded systems of interaction that affect children, thereby shaping their educational experiences. The implementation of children's rights is necessarily linked to understanding the diverse social conditions of childhood. It is essential to explicitly state these foundations to make visible the realities of migrant and refugee children in a reliable way ( Mayall, 2002). When discussing the rights of migrant and refugee children, it is crucial to consider their inclusion as full members of society with the same rights as non-migrant children. The intersection of their social identities as children and migrants can hinder their ability to equitably access educational opportunities and experiences ( Ulloa-Cortés, 2021). Although European policymaking places great emphasis on the integration of migrants and highlights equality and intercultural dialogue as the cornerstone principles for embracing the increasing diversity of European societies ( Council of Europe, 2008), migrant and refugee children still experience educational inequality ( European Commission/EACEA/Eurydice, 2019).

First, migrant and refugee children are especially vulnerable as their basic needs and personal development depend on the degree of recognition of their legal status and social rights by the host country. This recognition process is often influenced by the political will of those in power ( García Cívico, 2010). Rights and citizenship form the foundation for establishing expectations and obligations for the inclusion process ( Ager & Strang, 2004). Migrant children who access educational systems in vulnerable positions due to legal status, such as asylum-seekers, migrants in an irregular situation, and unaccompanied minors, are at a significantly higher risk of early school leaving and educational underachievement ( European Commission/EACEA/Eurydice *et al.*, 2019). Previous studies have classified these children's specific barriers into seven categories: curricular, teacher-related, structural aspects of the education system, socio-emotional factors, xenophobia and discrimination, socio-economic factors, values and cultural aspects ( Neubauer, 2019).

Secondly, there are also barriers at the meso level that may have a more limited range of influence. However, as this level represents children's interpersonal relationships and communities, it holds the potential for a more profound effect on children. Therefore, emphasis has been placed on children's participation in their educational community and schools as sources of psychosocial support that provide a safe context for development ( Vilela *et al.*, 2018).

Mainstream schools are formal educational settings that receive resources to provide all children with equitable quality education and promote their future learning opportunities. However, usually migrant and refugee children arrive in the host country with a pre-migration or transit experience that is detrimental to their mental health and general wellbeing that needs to be addressed in order for them to be able to take advantage of said opportunities at the same level as their non-migrant peers. Similarly, they encounter post-migration challenges that make it more difficult for them to engage in schools fully. For instance, they may experience psychological distress due to previous traumatic experiences, difficulties adjusting to a new language and culture, family separation, and discrimination ( Bronstein & Montgomery, 2011; Fazel *et al.*, 2012). Particularly, refugee children are at a greater risk of developing emotional and behavioural difficulties due to the multiple losses they experience ( Beiser & Hou, 2016).

To provide an answer to such needs, educational settings can incorporate social and emotional skills into school curricula to potentially enhance the resilience of migrant and refugee children and positively impact their well-being by promoting a culture of sharing their difficult experiences with others to ease the burden they represent ( Fazel & Betancourt, 2018). Furthermore, schools can provide a safe environment to address potential problems by offering *psychosocial support services*, such as counselling or therapy. Providing these services within the school can be particularly beneficial as families and children may be hesitant to attend external mental health services but may feel more comfortable accessing them at school ( Heidi *et al.*, 2011). Unfortunately, not all schools can hire sufficient work staff to provide this support as an integral service. A study conducted in six European countries found that around half of the participating schools do not have any staff to offer psycho-social support or personal counselling to students ( Martin *et al.*, 2023).

*Effective leadership* is crucial in schools for all children, and it can have major implications for culturally diverse students such as migrant and refugee children. Inclusive education demands culturally responsive leadership dedicated to achieving equity, inclusion, and equal opportunities for all students while embracing the local school culture's agendas ( Khalifa *et al.*, 2016). This can only be achieved through the commitment of school principals and management teams, collaborative teamwork among school staff, and their appropriate training in cultural responsiveness ( Shaw, 2017). These factors entail a shared framework among school staff that maintains a positive attitude towards students and their learning abilities ( Topping & Maloney, 2005). Adequate training of the teaching staff is critical for effective inclusive education, as teachers should possess sufficient knowledge about learning difficulties and skills to develop specific instructional methods ( Forlin, 2010). While the theory provides relevant evidence on the benefits of clear leadership, its practical application is not without challenges ( Nicholas *et al.*, 2021). The IMMERSE Project conducted in six European countries found that although most school principals declared that intercultural values as part of their syllabus were one of their insignias, less than half of the schoolteachers declared that intercultural values as part of their lessons were one of their insignias ( Martin *et al.*, 2023). This mismatch between principals’ declarations on their leadership around intercultural values and its practical application by teachers produces a disadvantage for migrant and refugee children. Additional previous research has also addressed the educational challenges of refugee students, focusing on online learning access and digital inequality and proposing policy improvements for post-pandemic education ( UNHCR, 2022). 

Besides, intensive *learning support* should be provided by schools to ease the cognitive and emotional challenges faced by newly arrived migrant and refugee children ( Sinkkonen & Kyttälä, 2014; Taylor & Sidhu, 2012). Preparatory classes are particularly important at the secondary level, where students face greater difficulties in acquiring a new language, and the curriculum demands language proficiency ( Crul *et al.*, 2019). However, extended preparatory classes could potentially hinder integration by separating migrant and refugee children from their native peers or delay other academic learning by focusing solely on language acquisition ( Nilsson & Bunar, 2016).

European countries have committed to implementing inclusive educational approaches. However, *physical and social segregation* often occurs due to housing segregation, which, in turn, leads to school segregation ( Agirdag *et al.*, 2011; Lindgren, 2010; Slee, 2012). Migrant students are particularly affected by the concentration of socio-economically disadvantaged neighbourhoods and schools with lower academic standards, which negatively impacts their educational achievement. Empirical studies have shown that children from ethnic and lower socio-economic minorities are disproportionately represented in special schools, where they often experience academic underachievement ( Kavale, 2007; Nusche, 2009). The findings suggest that inclusion in mainstream schools with additional resources can achieve better academic, social, and personal outcomes. However, better results are mainly determined by the quality of the provided resources ( Shaw, 2017).

For this reason, non-formal educational settings are particularly relevant for migrant and refugee children. These spaces provide alternative educational opportunities and offer more nuanced attention to their educational needs. They also create effective bridges and bonds with the community, which eases their social inclusion ( Wiktorin, 2017). In this respect, the Sirius report “Role of non-formal education in migrant and refugee children inclusion: links with schools” emphasises the importance of collaboration between formal and non-formal educational settings in promoting the overall development of children. This collaboration can help foster inclusion, well-being, and academic achievement ( Lipnickienė *et al.*, 2018). In fact, this report demonstrates that non-formal education can facilitate inclusion by bridging with formal education, combating segregation, providing academic and emotional support, offering linguistic support, overcoming trauma, building resilience, promoting intercultural competencies, and preventing radicalisation and violence. Despite the benefits, non-formal education is often less visible, less recognised, and harder to validate ( Souto-Otero & Villalba-Garcia, 2015).

In conclusion, negative attitudes towards migrants and refugees can hinder their ability to have an optimal educational experience. This can have an impact on both macro and meso levels, ultimately affecting the migrant children themselves. Discrimination experiences can significantly alter the attitudes and orientations of migrants and refugees towards mainstream society, which can reduce their motivation to integrate into the host culture ( Lutterbach & Beelmann, 2021). Additionally, bullying or discriminatory treatment by peers, whether from the host society or their own ethnic group, can have devastating consequences on the relationship between migrant and refugee children and their peer group ( Kang, 2010).

## A discussion of socio-educational inclusion for migrant and refugee children

As explained in the previous section, children's educational experiences are complex and involve multiple parties and key figures influencing how children engage with, feel about, and develop their learning trajectories ( Ballaschk & Anders, 2020). The barriers and gaps to socio-educational inclusion discussed in this text represent significant obstacles to the learning and participation of migrant and refugee children. These obstacles are based on the different identities and social labels that intersect with them. To solve these problems, it is necessary to adopt an inclusive and intercultural approach to education that prioritises children's social and personal needs. This aligns with the Incheon Framework and strategies for sustainable development and education ( UNESCO, 2016).

The strategy for future actions in educational inclusion and equity development stresses the need to ensure access to education for all students, particularly those in vulnerable situations, and to strengthen the quality of learning and skills ( UNESCO, 2016). Therefore, in order to address the educational challenges faced by migrant and refugee children, it is necessary to remove barriers to education by effectively implementing inclusive education. Additionally, it is important to be aware of the inequalities arising from a different socio-cultural context, which can be achieved by adopting an intercultural model for educational inclusion ( IOM, 2019).

Hereafter, we will discuss these concepts in-depth to offer insight into a child-centred definition of socio-educational inclusion for migrant and refugee children that embodies inclusive interculturalism in education.

### An analysis of the contribution of intercultural education

The intercultural approach in education represents a pedagogical framework designed to facilitate an understanding, respect, and appreciation of cultural diversity ( Sales *et al.*, 2023). Derived from the necessity to address cultural discrepancies in societies that are becoming increasingly diverse, this approach encourages the implementation of inclusive practices that acknowledge and value students' cultural backgrounds. It confers benefits upon the field of education by fostering a more inclusive and equitable learning environment, enhancing students' social skills, and preparing them for global citizenship ( Mitchell & Paras, 2018). The intercultural approach has been operationalised in educational settings by implementing curricula responsive to cultural contexts, providing training programmes for educators, and establishing policies that support diversity and inclusion ( Portera, 2020).

Interculturalism is a crucial model for inclusive education that concentrates on developing cultural competencies to promote peacebuilding and understanding among students from diverse backgrounds. This approach goes beyond passive coexistence and aims to establish a sustainable way of living together in multicultural societies through mutual understanding, respect, and dialogue between different cultural groups ( UNESCO, 2010). The UNESCO’s landmark document *Guidelines on Intercultural Education* ( UNESCO, 2006) states that applying an intercultural approach to educational contexts requires respecting the cultural background of all students and providing them with culturally appropriate and responsive quality education. To achieve this, students must be provided with cultural knowledge and taught to develop positive cultural attitudes and skills that enable them to intentionally recognise identities as multiple, hybrid, and complex. This will help them avoid categorisations and develop solidarity and respect towards diverse groups of people ( Belmonte, 2017).

Removing barriers to the education of migrant and refugee children requires fostering intercultural dialogue within school cultures. The increasingly diverse student bodies in educational settings are a current concern that impacts the school climate and the psychosocial well-being of both migrant and non-migrant background students ( OSCE Office for Democratic Institutions and Human Rights (ODIHR), 2018). Intercultural dialogue in this context facilitates the exchange of perspectives and knowledge to achieve mutual understanding and respect ( Council of Europe, 2008). Therefore, intercultural dialogue is a crucial tool for promoting more inclusive educational environments for migrant and refugee children.

Introducing children to core values, attitudes, and skills and encouraging their adoption beyond school settings is fundamental for developing intercultural competence. This vision motivates children to embrace their differences and similarities with others, creating a solid foundation for respect and creating more inclusive and cohesive societies ( Essomba, 2014). In this sense, intercultural education is child-centred, highlighting the role of children as active participants and shapers of their own realities. It emphasises that children are reliable experts and acknowledges their influence not only on their own lives but also on the lives of those around them and, ultimately, on society ( Aguado-Odina & Sleeter, 2021).

As the specialised literature states, intercultural education is a form of educational inclusion and is considered one of the best ( Arroyo, 2013). The guiding principle of inclusive education is equity. Attention is paid to the particular educational needs of students, seeking to unveil how they are socially conditioned. From this perspective, intentional efforts are made to reverse inequality and enhance students' competence to develop and function across diverse sociocultural environments. In this context, equality refers to the availability of opportunities to make informed choices and access social, economic, and educational resources. The international consensus is to shift the focus from diverse students to local mainstream schools ( Ainscow, 2020). From this perspective, inclusion is about creating school cultures that encourage the reassessment of learning conditions offered to students. This includes identifying specific barriers to participation and learning, fostering collaboration among all members of the educational community, sharing practices and ideas, promoting innovation, building upon previous knowledge and practice, and making use of available resources for learning support ( Ainscow & Sandill, 2010).

Therefore, it is important to recognise the systems that directly impact children's adaptation to develop intercultural competence. Migrant and refugee families are rooted in cultural and social contexts that differ significantly from those of native families, resulting in the formation of distinct identities. Inclusive intercultural education involves adopting a pluralistic perspective that acknowledges the diversity of experiences and benefits all students ( Moser *et al.*, 2017).

### Inclusive interculturalism: Addressing the socio-educational shortcomings of migrant and refugee children in Europe 

The previous sections have shown that a multidimensional perspective is necessary for accurately understanding the socio-educational inclusion of migrant and refugee children. In this manner, the model of inclusive interculturalism is proposed as a child-centred conceptual framework to address the socio-educational inclusion of migrant and refugee children. This model is designed with a child-centred approach, emphasising the importance of closely aligning with the child's needs and factors influencing future development. The literature identifies five dimensions of socio-educational inclusion ( Serrano Sanguilinda *et al.*, 2019): legal status, linguistic competencies, psycho-social well-being and health, social relations, and educational achievements. The barriers presented in the previous section influence the children’s results in these dimensions. Considering this conceptual framework can help develop a more accurate child-centred view of the socio-educational inclusion of migrant children, addressing gaps that have been identified. Additionally, considering the children's perspective when examining these dimensions may advance migration studies, which have traditionally focused on adults.

Attention to migrant children's *access to rights* reveals that their basic needs, personal development, and social inclusion depend primarily on the degree of recognition given to their migratory status by the host society and the set of rights, expectations, and obligations attributed to it. This also depends on the prevailing idea of citizenship ( Ager & Strang, 2004; Bauböck, 2003). From a child-centred perspective, this dimension of integration relies on the fundamental supporting principle that international and EU legislation prioritises children's status over migrants in defining and attributing a set of rights. These rights include those enshrined in the United Nations Convention on the Rights of the Child. This set includes the right to access education, healthcare, and other fundamental rights. State authorities must always prioritise the child's best interest in any administrative decision ( UNHCR, 2008). However, to understand this dimension from a child's perspective, it is important to consider the various situations and ways in which migrant children may be affected by external variables, such as national legislation on migration and citizenship, as well as personal variables, such as family migration history, origin, and age, which may impact their access to rights. This is a significant issue, particularly in the case of unaccompanied minors. Age determination practices often categorise them as adults or nearly adults, breaching their children’s rights and hindering their integration paths. Also, previous research has pointed to policies permitting “warm returns” of these children and facilitating their mass deportation as key issues for the settlement of unaccompanied minors in Europe ( Francia *et al.*, 2021).

Acquiring *linguistic competence* in the host society's national language is a crucial integration aspect. Communicating allows individuals to engage in social networks, participate in civic rights and develop an individual and collective identity. As Heckmann (2008) has demonstrated, factors such as a young age, a solid foundation in a mother tongue, a certain degree of family cultural capital, and the provision of language classes by schools are essential in achieving this goal. The CCA recommends emphasising the significance of linguistic competence in ensuring children's autonomy, voice (i.e. the expression of their views and needs), and social participation ( Johnson & Johnson, 2016). Additionally, it is important to incorporate children's perspectives when addressing language issues in formal and informal educational settings. Educators should recognise and value children's competencies in their first language and promote multilingualism as a tool for educational inclusion and intercultural education. UNESCO has advocated for home language teaching in pre-primary and primary education since 1953. Scientific research has consistently highlighted the positive effects of such teaching on students' social, cognitive, and linguistic development ( European Commission/EACEA/Eurydice, 2019).

The primary outcomes of integration are *health and psychosocial well-being*. Integration can be interpreted as a process of social inclusion that helps individuals meet their basic needs ( Harttgen & Klasen, 2009). The relationship between integration and well-being is particularly evident when child-centred perspectives are adopted instead of adult-centric approaches. The assessment of migrant children's integration, particularly in connection with the social dimension, highlights the importance of aspects such as children's happiness, self-esteem, and sense of belonging ( Bajo Marcos *et al.*, 2022). Besides, the literature widely studies the areas where well-being and group identity overlap. Positive feelings of belonging may help counteract the negative consequences of outside threats ( Liu & Zhao, 2016) and may play a key role in maintaining psychological health ( Eccles *et al.*, 2006). The CCA aims to expand on this concept by analysing the agency and resilience of migrant children ( Fonagy *et al.*, 1994) in host societies. It is important to consider age, as adolescence is a particularly vulnerable stage in emotional development, during which migrant children may experience feelings of vulnerability, exclusion, and lack of confidence ( Perez, 2016). Child-accessible psycho-social support measures, such as counselling or therapeutic services at schools, can enable children to take an active role in caring for their own mental health. This is particularly important in cases where accessing health services for children and families is critical. Teaching social and emotional competencies, creating inclusive and integrative climates at school, and promoting social connectedness with both peers and teachers can improve migrant students' resilience and positively impact their academic outcomes ( European Commission/EACEA/Eurydice, 2019).

*Social relations*, including bonds, bridges, and links, form the structural foundation of social capital. They serve as pathways for the transmission of values, attitudes, and behaviours ( McLeod & Lively, 2003). Social bonds with peers and teachers, as well as links with institutions, are particularly important outcomes of migrant children's integration. However, factors such as low family status can affect trust in institutions and the size of the school network among migrant students ( Santagati, 2015). Non-inclusive school curricula and climates ( Agirdag *et al.*, 2011), as well as the spread of negative societal representations and prejudices towards migration, can also be barriers to integration ( Banks, 1995). The CCA framework highlights the role of children as competent social actors who can participate in decision-making regarding their social lives ( James & Prout, 1997). Additionally, the focus on agency leads to a non-essentialist view of children's cultural identities as always hybrid and negotiated within continuous interactions with groups ( Baraldi, 2021). In a CCA, it is crucial to ensure that children who are at high risk of exclusion actively participate in their daily interactions. This requires recognising diversity as a positive value, particularly in educational settings, and actively contributing to creating pluralist cultures where all children, regardless of their origin, culture, ethnicity, beliefs, gender, etc., are included and can participate in the community.

Finally, children with migrant backgrounds face significant challenges and difficulties, and they systematically achieve lower *academic results* than native children without an immigrant background ( OECD, 2016). Academic success depends on various situational factors, including psychological well-being, emotional development, language acquisition, and relevant social factors. Family cultural capital plays a crucial role in shaping motivation and aspirations. The influence of a migrant background on students' learning opportunities appears to be more dependent on socio-economic status than the migration process itself ( European Commission/EACEA/Eurydice, 2019). Successful socioeconomic integration of parents reduces the risk of drop-out and educational segregation in vocational schools. The CCA highlights the importance of involving parents in formal and non-formal educational institutions and encouraging children to participate actively in educational communities.

The outcomes mentioned are complex and multidimensional. Therefore, it is necessary to consider different levels of analysis for each of them. Furthermore, their various components and determinants are interrelated. For example, well-being outcomes significantly impact social relations, and both, in turn, affect academic achievement. Finally, the processes mediating inclusion outcomes through facilitators and barriers take place and can be observed in different settings within the social system. The CCA emphasises the need for rigorous consideration and in-depth empirical exploration of the first-hand experiences of migrant children and their understanding of the integration process.

## Conclusions

This narrative review presents a theoretical discussion of the open debates surrounding the specific challenges experienced by migrant and refugee children in education. It explores how their socio-educational inclusion may help to alleviate these shortcomings. The first section reviews child-centred education and the limited development of conceptual work and guiding methodologies for migrant and refugee students. Without age-appropriate and culturally sensitive theoretical frameworks that acknowledge the social conditions and subjectivities of migrant and refugee children, empirical research aimed at evidence-based educational practices is flawed. In addition, as migrants and refugees, these children experience barriers to their inclusion in education, preventing them from fully engaging and benefiting from it. Finally, we examine the barriers that affect migrant and refugee children's access to child-centred education and we explore how macro and meso level factors have both proximal and distant effects on this issue.

Reflecting on these challenges, we propose inclusive interculturalism as a child-centred approach to address the socio-educational shortcomings of migrant and refugee children in European educational settings. This text presents the positive contribution of intercultural education to children's development. It highlights the growth of cultural awareness and intercultural skills, as well as the promotion of children's agency through the underlying values of intercultural education. In conclusion, we examine the various aspects of socio-educational inclusion for migrant and refugee children. We connect the narratives of migration and childhood studies to identify new research areas that aim to reduce educational inequalities experienced by these children. This theoretical discussion can serve as a foundation for designing intervention models for child-centred education of migrant and refugee children in future works.

Finally, while this study provides a comprehensive narrative overview of the socio-educational integration of migrant and refugee children, it has several limitations. First, the use of secondary sources limits the ability to capture real-time data and specific contextual factors affecting the education of migrant and refugee children. This reliance on existing literature may miss emerging trends or issues that could provide additional insight into current challenges. Secondly, the focus on European education systems may limit the generalisability of the findings to non-European contexts where different policies, cultural dynamics and integration frameworks prevail. Finally, the conceptual nature of the study means that practical interventions are suggested rather than empirically tested, leaving a gap between theory and practice that future research should address through empirical studies or case-based evaluations of proposed frameworks.

## Ethics and consent

Ethical approval and consent were not required.

## Data Availability

No data are associated with this article.

## Footnotes

^1^From an inclusivist perspective, a migrant is defined as an individual who is either in the process of moving or has already relocated across an international boundary or within the same nation, away from their usual residence. This definition applies irrespective of whether the migration is voluntary or forced, the reasons behind it, the legal standing of the individual, or the duration of their stay in the new location ( UNHCR UNICEF, & IOM, 2019).

^2^A refugee is “a person who, owing to a well‐founded fear of persecution for reasons of race, religion, nationality, membership of a particular social group or political opinion, is outside the country of his nationality and is unable or, owing to such fear, is unwilling to avail himself of the protection of that country; or who, not having a nationality and being outside the country of his former habitual residence as a result of such events, is unable or, owing to such fear, is unwilling to return to it” ( UNHCR, 2010).

^3^In this text, the terms "integration" and "inclusion" are used to emphasise different facets of the processes by which individuals enter mainstream contexts. We use “integration” in line with migration studies literature when the focus is put on how individuals resettle and become full members of existing social structures. Instead, we use inclusion in line with education literature when the focus is put on transforming these structures to accommodate the diverse needs of all individuals. In this sense, previous literature has considered that integration can be seen as a preliminary step to inclusion ( Slee, 2012).
