# Supplementary material for: A child-centred intercultural approach to the socio-educational inclusion of migrant and refugee children
Source: Open Res Eur. 2024 Oct 17;3:220. Originally published 2023 Dec 29. [Version 2] doi: 10.12688/openreseurope.16999.2 (PMC11401985; doi:10.12688/openreseurope.16999.2)
Supplement: Peer review history

# Reviewer report: Reviewer response for version 2

**DOI:** 10.21956/openreseurope.20062.r45220

**Published:** 2024-10-24

**Authors:** Adrián Neubauer

**Affiliations:** 1 Department of Education, Universidad a Distancia de Madrid, Collado Villalba, Community of Madrid, Spain

Authors have made the suggested changes. Thank you for taking all comments into consideration.

Are all factual statements correct and adequately supported by citations?

Partly

Is the review written in accessible language?

Yes

Are the conclusions drawn appropriate in the context of the current research literature?

Partly

Is the topic of the review discussed comprehensively in the context of the current literature?

Partly

Reviewer Expertise:

Educational policy

I confirm that I have read this submission and believe that I have an appropriate level of expertise to confirm that it is of an acceptable scientific standard.

# Reviewer report: Reviewer response for version 1

**DOI:** 10.21956/openreseurope.18370.r37719

**Published:** 2024-09-14

**Authors:** Adrijana Visnjic-Jevtic

**Affiliations:** 1 University of Zagreb, Zagreb, Croatia

The rising phenomenon of children's migration, be it coerced or voluntary, poses a challenge to policies striving for social cohesion and the protection of children's rights. The importance of a child-centric approach to the socio-educational inclusion of migrant and refugee children can contribute to the establishment of cohesive and inclusive societies therefore it is necessary to discuss and research the above mentioned. This review delves into the challenges associated with adopting a child-centric approach to the education of migrant and refugee children. It puts forth inclusive inter-culturalism as a child-centric strategy to address the socio-educational deficiencies experienced by these children in European educational settings.

 Anyway, the text effectiveness is constrained by the absence of contemporary literature, limiting insights into current trends and challenges surrounding the child-centric approach in the education of migrant and refugee children. Incorporating more recent sources is imperative to capture evolving policies and to assess the real-time implications for social cohesion and children's rights. So, I would suggest to find recent publications if there are any understanding and addressing the socio-educational inclusion of migrant and refugee children through a child-centric approach is of paramount importance in fostering cohesive and inclusive societies. This topic holds significance as it directly influences the well-being and development of vulnerable populations, shaping their access to fundamental rights and opportunities. A comprehensive exploration of this subject not only contributes to academic discourse but also informs policy and practice, playing a crucial role in creating educational environments that promote diversity, equity, and social harmony.

Are all factual statements correct and adequately supported by citations?

Yes

Is the review written in accessible language?

Yes

Are the conclusions drawn appropriate in the context of the current research literature?

Yes

Is the topic of the review discussed comprehensively in the context of the current literature?

Partly

Reviewer Expertise:

Early childhood education; teacher profession

I confirm that I have read this submission and believe that I have an appropriate level of expertise to confirm that it is of an acceptable scientific standard, however I have significant reservations, as outlined above.

# Response

**Published:** 2024-10-03

**Authors:** Ángela Ordóñez-Carabaño

**Affiliations:** Psychology, Comillas Pontifical University, Madrid, Community of Madrid, Spain

Dear Dr. Visnjic-Jevtic, We would like to sincerely thank you for your thoughtful and constructive feedback on our manuscript. We have carefully considered your comments and have made the necessary revisions. Below is a summary of the changes made:

- **Incorporation of Recent Literature**:
  - We have updated the manuscript by incorporating more recent and relevant sources to provide a more comprehensive analysis of current policies and challenges surrounding the child-centred approach in the education of migrant and refugee children.
- **Expanded Discussion on the Relevance of the Topic**:
  - We have expanded our exploration of how a child-centred approach can enhance the socio-educational inclusion of migrant and refugee children.
- **Contribution to Practice**:
  - We discuss how the findings can help create educational environments that promote diversity, equity, and social harmony.

We hope that these revisions address your concerns and improve the quality and clarity of the manuscript. Thank you again for your valuable feedback.

# Reviewer report: Reviewer response for version 1

**DOI:** 10.21956/openreseurope.18370.r39182

**Published:** 2024-08-21

**Authors:** Adrián Neubauer

**Affiliations:** 1 Department of Education, Universidad a Distancia de Madrid, Collado Villalba, Community of Madrid, Spain

Dear authors:

 Here are a few comments on the article:

 - Firstly, the abstract should follow the "Introduction. Objective. Method. Results. Discussion".

 - On the other hand, the writing is nice and clear.

 - Regarding the methodological part, the article does not detail the search process and the selection of the sources analysed. It would be advisable to design the study according to a systematic review where the inclusion and exclusion criteria are detailed (e.g. language, date of publication, type of source...). The lack of systematisation in the search and selection of sources may bias the results obtained. 

 - In line with the above, numerous studies have been developed in recent years that have analysed the right to education of immigrant children and/or interculturality in the European Union and which have not been covered in this article. To name but a few, we recommend reviewing the INNO4DIV project [Ref 3] & [Ref 2] and some authors (e.g. Jordi Pamiès [ref 4] &[ref 5], Adrián Neubauer ,et.al., [Ref 1] & [Ref 6]].

 - The conclusions presented are too brief and are not put in dialogue with other authors.

 - Finally, future lines of research are included, but not the limitations of the study itself.

 I hope these comments will help you to strengthen your work in the future.

Are all factual statements correct and adequately supported by citations?

Partly

Is the review written in accessible language?

Yes

Are the conclusions drawn appropriate in the context of the current research literature?

Partly

Is the topic of the review discussed comprehensively in the context of the current literature?

Partly

Reviewer Expertise:

Educational policy

I confirm that I have read this submission and believe that I have an appropriate level of expertise to state that I do not consider it to be of an acceptable scientific standard, for reasons outlined above.

## References

[1] : El derecho a la educación de los menores refugiados y solicitantes de asilo desde el enfoque basado en los derechos humanos: dificultades, finalidad e intervención educativa. *Revista Española de Educación Comparada* .2019; 10.5944/reec.35.2020.24342 10.5944/reec.35.2020.24342

[2] : Child-Centred Teaching: Helping Each Child to Reach Their Full Potential. *Education Sciences* .2021;11(6) : 10.3390/educsci11060280 10.3390/educsci11060280

[3] : Addressing educational needs of teachers in the EU for inclusive education in a context of diversity (INNO4DIV Project). *Publications Office of the European Union* . 10.2760/857350 10.2760/857350

[4] : Inclusive education: Open debates and the road ahead. *SpringerLink* . 10.1007/s11125-009-9129-7 10.1007/s11125-009-9129-7

[5] : Feminism, Islam and higher education: towards new roles and family relationships for young Spanish-Moroccan Muslim women in Spain. *Race Ethnicity and Education* . 10.1080/13613324.2021.1890565 10.1080/13613324.2021.1890565

[6] : Unaccompanied Migrant Children’s Rights: A Prerequisite for the 2030 Agenda’s Sustainable Development Goals in Spain and Sweden. *Social Sciences* . 10.3390/socsci10060185 10.3390/socsci10060185

# Response

**Published:** 2024-09-10

**Authors:** Ángela Ordóñez-Carabaño

**Affiliations:** Psychology, Comillas Pontifical University, Madrid, Community of Madrid, Spain

Dear Dr. Neubauer, We greatly appreciate the time and effort you have taken to review our work. We are pleased to inform you that we have carefully considered each of your suggestions and incorporated the necessary changes in the revised manuscript. Below is a summary of the revisions that have been made.

- **Abstract structure**: We have modified the abstract to follow the recommended structure. **Methodology:** We have included a detailed description of our narrative review in the methodology section. We have included the term "narrative" to avoid confusion in the manuscript. This method allowed us to review a broader range of studies and offer an overarching summary, along with interpretation and critique. Moreover, it provided us with the flexibility to incorporate diverse viewpoints and leverage the unique perspectives of the review team, which influence the analysis. In case you require further information, you can consult the following article: Sukhera, J. (2022). Narrative Reviews: Flexible, Rigorous, and Practical. *Journal of Graduate Medical Education, 14*(4), 414–417. https://doi.org/10.4300/JGME-D-22-00480.1
- **Literature review:** We reviewed and included recent studies analysing the right to education of immigrant children and/or interculturality in the European Union, including the INNO4DIV project and the work of the authors you mentioned.
- **Discussion:** We have expanded the discussion.
- **Limitations:** We have the limitations of our study in the conclusions section. We believe that these revisions have strengthened the manuscript and hope that it now meets your expectations.

Thank you again for your valuable feedback.

# Reviewer report: Reviewer response for version 1

**DOI:** 10.21956/openreseurope.18370.r39185

**Published:** 2024-08-14

**Authors:** Lucija Dežan, Maja Zadel

**Affiliations:** 1 Institute for Social Studies,, Science and Research Centre Koper, Koper, Slovenia; 2 Institute for Social Studies, Science and Research Centre Koper, Koper, Slovenia

First of all, we would like to thank for the opportunity to review this manuscript. The article titled "A child-centred intercultural approach to the socio-educational inclusion of migrant and refugee children" explores an important and critical issue that is relevant for the global community as well as educational practitioners. Its central point - promoting a child-centred intercultural approach as a vehicle for integration - holds significant implications for policy development as well for institutional support structures. Nevertheless, the article requires considerable changes to effectively and adequately present the topic.

 Initially, it is advisable to clearly define the key terms utilized within the manuscript (e.g. distinction between migrant and refugee children, the concepts of integration and inclusion, the criteria for successful integration, and what constitutes capacity building). Once the terms are defined, they should be consistently used to ensure textual coherence. For example, the interchange or perceived opposition between 'inclusion' and 'integration' at various points introduces ambiguity. Furthermore, the manuscript would benefit from an explicit definition on 'inclusive interculturalism' and its connection to a child-centred approach (CCA). Although the article eventually references some intersections related to rights access and health & psychosocial well-being, a definitive linkage between inclusive interculturalism and CCA is not present. Similarly, the mechanisms by which CCA (or inclusive interculturalism) enhances the resilience of migrant and refugee children require further clarification.

 Moreover, authors should reconsider whether the aim of this article is really to discuss the challenges of adopting a child-centred approach to education for migrant and refugee children. At times, the manuscript ambiguously addresses whether certain elements are prerequisites for or outcomes of the discussed approaches. The narrative occasionally overlooks negative aspects within the educational setting that may impede integration and interculturalism.

 Regarding methodology, a detailed description of the literature review process, including the databases consulted and the inclusion/exclusion criteria established, would substantially enrich the article. 

 Lastly, the manuscript contains repetitive statements (e.g. Migrant and refugee children are especially vulnerable as their basic needs and personal development depend on the degree of recognition of their legal status and social rights by the host country.«), typos (Effective leaderships crucial in schools.) and editing notes (The text is clear, concise, and uses objective language with a formal register. The grammar, spelling, and punctuation are correct.).

Are all factual statements correct and adequately supported by citations?

Yes

Is the review written in accessible language?

Partly

Are the conclusions drawn appropriate in the context of the current research literature?

Partly

Is the topic of the review discussed comprehensively in the context of the current literature?

Partly

Reviewer Expertise:

Migrant studies, integration process, migrant learners

We confirm that we have read this submission and believe that we have an appropriate level of expertise to confirm that it is of an acceptable scientific standard, however we have significant reservations, as outlined above.

# Response

**Published:** 2024-09-10

**Authors:** Ángela Ordóñez-Carabaño

**Affiliations:** Psychology, Comillas Pontifical University, Madrid, Community of Madrid, Spain

Dear Dr. Dežan and Dr. Zadel, We greatly appreciate the time and effort you have taken to review our work and provide constructive comments. We are pleased to inform you that we have carefully considered each of your suggestions and incorporated the necessary changes in the revised manuscript. Below is a summary of the revisions that have been made: Conceptual clarification:

- We have clearly defined key terms, including the distinction between migrant and refugee children, the concepts of integration and inclusion, the criteria for successful integration and what constitutes capacity building.
- These terms have been used consistently throughout the manuscript to ensure coherence.
- We have provided an explicit definition of 'inclusive interculturalism' and clarified its link to the child-centred approach (CCA).

- We have clarified how both the child-centred approach and inclusive interculturalism enhance the resilience of migrant and refugee children.
- We have explicitly stated that the aim of the article is to explore the challenges of adopting a child-centred approach to education for migrant and refugee children in Europe and propose a framework for inclusive interculturalism that addresses these challenges.
- We have clarified any ambiguity about whether certain elements are prerequisites or outcomes of the approaches discussed.
- We have considered and included discussions on potential negative aspects within the educational setting that may hinder integration and interculturalism.

Methodology:

- We apologise for the misunderstanding as this article is not a systematic review, but rather a narrative review. We have included the term "narrative" to avoid confusion in the manuscript. This method allowed us to review a broader range of studies and offer an overarching summary, along with interpretation and critique. Moreover, it provided us with the flexibility to incorporate diverse viewpoints and leverage the unique perspectives of the review team, which influence the analysis. In case you require further information, you can consult the following article: Sukhera, J. (2022). Narrative Reviews: Flexible, Rigorous, and Practical. *Journal of Graduate Medical Education, 14*(4), 414–417. https://doi.org/10.4300/JGME-D-22-00480.1

Editing and Writing:

- We have removed repetitive statements, corrected typographical errors and addressed the editorial notes.

We hope that these revisions will meet your expectations and improve the overall quality and clarity of our manuscript.

# Reviewer report: Reviewer response for version 1

**DOI:** 10.21956/openreseurope.18370.r39186

**Published:** 2024-04-22

**Authors:** Magda Vitsou

**Affiliations:** 1 University of Thessaly, Thessaly, Greece

This review articulates a compelling argument for adopting a child-centered approach to education for migrant and refugee children, emphasizing the importance of inclusive interculturalism in fostering social cohesion and protecting children's rights. The authors present a well-rounded perspective on the challenges faced by these children and propose practical solutions rooted in inclusive educational practices. However, integrating recent references and reports (post-2019) into the review will enhance its academic credibility and relevance. 

 One strength of this review is its emphasis on collaboration among children, educators, communities, and local institutions to create inclusive educational environments. This collaborative approach is essential for promoting participation and ensuring that migrant and refugee children feel a sense of belonging within their new communities.

 Furthermore, the focus on child-centred practices to enhance intercultural and linguistic competences, as well as children's agency, is commendable. By prioritizing children's rights, social and emotional well-being, and recognition of cultural diversity, the proposed approach seeks to empower these children and facilitate their integration into society.

 The review also rightly highlights the pivotal role of schools in integrating migrant and refugee children. Schools not only provide academic education but also serve as crucial platforms for social integration and intercultural understanding. The emphasis on reducing inequalities and fostering equitable societies through inclusive education is both timely and necessary.

 Overall, this review offers a positive and forward-thinking perspective on addressing the socio-educational challenges faced by migrant and refugee children in Europe. By advocating for child-centered policies and practices rooted in inclusive interculturalism, the review contributes valuable insights to the discourse on social inclusion and education. 

Global education monitoring report, 2020: Inclusion and education: all means all | UNESCO

EJ1100968.pdf (ed.gov)

Guide for the development and implementation of curricula for plurilingual and intercultural education - Language policy (coe.int)

Document - Education on hold: Education Policy Brief (September 2023) (unhcr.org)

Europe: Education policy brief | Global Focus (unhcr.org)

Are all factual statements correct and adequately supported by citations?

Yes

Is the review written in accessible language?

Yes

Are the conclusions drawn appropriate in the context of the current research literature?

Yes

Is the topic of the review discussed comprehensively in the context of the current literature?

Partly

Reviewer Expertise:

Interculturalism, inclusive education, early childhood education, drama, and puppetry in education, migrant and refugee children

I confirm that I have read this submission and believe that I have an appropriate level of expertise to confirm that it is of an acceptable scientific standard.

# Response

**Published:** 2024-09-10

**Authors:** Ángela Ordóñez-Carabaño

**Affiliations:** Psychology, Comillas Pontifical University, Madrid, Community of Madrid, Spain

Dear Dr. Vitsou, Thank you very much for your detailed and constructive feedback on our manuscript. We appreciate the time and effort you have taken to provide us with insightful comments. We are pleased to inform you that we have introduced your suggested references and reports and other recent literature into the review. Thank you again for your valuable feedback.

# Reviewer report: Reviewer response for version 1

**DOI:** 10.21956/openreseurope.18370.r37208

**Published:** 2024-02-13

**Authors:** Linda Mitchell, Raella Kahuroa

**Affiliations:** 1 The University of Waikato, Hamilton, Waikato, New Zealand; 2 Faculty of Education, The University of Waikato, Hamilton, Waikato, New Zealand

This article is concerned with addressing barriers faced by migrant and refugee children in ‘integrating’ into a new country and the positive role that can be played by schools when they take a child-centred approach. Based on a review of literature, the authors propose ‘inclusive intercultural education as a child-centred approach to address the socio-educational deficits of these children in educational settings in Europe’. Educational institutions are often the first institution in a new country that children from refugee and immigrant backgrounds encounter. The topic of inclusion of refugee and immigrant children through education is therefore important, particularly given the challenges faced in resettlement and the current refugee and immigrant crisis. According to the UN Refugee Agency (UNHCR), 108.4 million people were forcibly displaced at the end of 2022, many of whom were young children. The rationale for the literature review and its framing around a ‘child-centred approach’ based on rights is sound.

 However, definitions and analysis warrant further attention. We suggest that the meanings of ‘refugee’ and ‘immigrant’ are defined, and that discussion of barriers and challenges faced by children from each of these backgrounds are separated since experiences may be very different. The meaning of a child-centred approach could also be explored more deeply. A ‘child-centred approach’ is defined as centring ‘on designing educational practices that prioritise the child over the school curriculum. The focus is on learning methods rather than the content to be covered, adapting to the abilities and needs of the children’. We were puzzled by this description since an inclusive curriculum will recognise each child’s rights and enable their participation – it is not a matter of prioritising one over the other.

 The article is described as a review of literature. Details need to be provided of the data bases searched and criteria for inclusion and exclusion of publications. The conceptual frame used to select and analyse articles could be expressed more succinctly. The writers drew on ecological theory and ideas of children as active participants and rights holders. It was argued that a child-centred approach is primarily ‘a theoretical approach’ and that ‘evidence and practical guidelines for its practical implementation are still insufficiently developed’. The extensive literature using multimodal methods to listen to children’s views, such as in the work of Alison Clark (Clark, 2004, 2023) with young children and of Caitlin Nunn (Nunn, 2018, 2022) with young people from refugee backgrounds counter this claim and offer valuable insights.

 Despite drawing on an ecological framework, the article pays little attention to the cultural, social and linguistic funds of knowledge that children from refugee and immigrant backgrounds bring with them, and their sense of place in home countries. The contribution from children and families to the curriculum in this respect could be a powerful source of enrichment for all children and teachers. Instead, the article is predominantly concerned with children’s ‘integration’ into a new society and portrays a slightly deficit view in its proposal for an approach ‘to address the socio-educational deficits of these children in European educational settings’.

 We think the discussion of an intercultural approach is the main contribution made through this article. The explanation of such an approach, its origins and why it is of benefit could be strengthened through reference to literature, including how it has been used in educational settings. Useful journal sources could be *Intercultural Education* and journals of intercultural studies.   

 There is some repetition throughout the article that distracts the reader from the main focus. There seem to be editing notes in the conclusion; these should be removed for clarity, and the whole article carefully proofread as there were a few minor editing/proofreading errors.

Are all factual statements correct and adequately supported by citations?

Partly

Is the review written in accessible language?

Yes

Are the conclusions drawn appropriate in the context of the current research literature?

Partly

Is the topic of the review discussed comprehensively in the context of the current literature?

Partly

Reviewer Expertise:

Early childhood education (ECE); ECE policy; culturally responsive pedagogy; refugee resettlement

We confirm that we have read this submission and believe that we have an appropriate level of expertise to state that we do not consider it to be of an acceptable scientific standard, for reasons outlined above.

## References

[1] The Mosaic Approach and research with young children/authors.2004;

[2] : Slow Knowledge and the Unhurried Child Time for Slow Pedagogies in Early Childhood Education. *Routledge* .2022;

[3] : Dispersed Belongings: A participatory arts-based study of experiences of resettled refugee young people in regional cities in Australia and the United Kingdom. A report for project partners. *e-space* .2018;

[4] : The participatory arts-based research project as an exceptional sphere of belonging. *Qualitative Research* .2022;22(2) : 10.1177/1468794120980971 251-268 10.1177/1468794120980971

# Response

**Published:** 2024-09-10

**Authors:** Ángela Ordóñez-Carabaño

**Affiliations:** Psychology, Comillas Pontifical University, Madrid, Community of Madrid, Spain

Dear Dr. Mitchell and Dr. Kahuroa, Thank you very much for your detailed and constructive feedback on our manuscript. We appreciate the time and effort you have taken to provide us with insightful comments. We are pleased to inform you that we have introduced each of the suggested changes to improve the quality and clarity of our manuscript. Below is a summary of the revisions made:

- We have defined the terms ‘refugee’ and ‘immigrant’ as a footnote to clarify them.
- We apologize for the misunderstanding as this article is not a systematic review, but rather a narrative review. We have included the term "narrative" to avoid confusion in the manuscript. This method allowed us to review a broader range of studies and offer an overarching summary, along with interpretation and critique. Moreover, it provided us with the flexibility to incorporate diverse viewpoints and leverage the unique perspectives of the review team, which influence the analysis. In case you require further information, you can consult the following article: Sukhera, J. (2022). Narrative Reviews: Flexible, Rigorous, and Practical. *Journal of Graduate Medical Education, 14*(4), 414–417. https://doi.org/10.4300/JGME-D-22-00480.1
- We provided details of the databases searched and the criteria for inclusion and exclusion of publications.[ÁO1] [EH2] 
- We detailed the conceptual framework used to select and analyze articles.
- We have clarified some of the barriers and challenges faced by refugee children specifically.
- We have detailed the meaning of a ‘child-centered approach’.
- We clarified that a child-centered approach involves not just prioritizing the child over the school curriculum but includes an inclusive curriculum that recognizes each child's rights and enables their participation.
- We reviewed and modified the claim regarding the theoretical nature of the child-centered approach, acknowledging the extensive literature on multimodal methods for listening to children's views.
- We included information on the cultural, social, and linguistic knowledge that children from refugee and immigrant backgrounds bring, as well as their sense of belonging in their home countries.
- We included how the contributions from children and families to the curriculum can enrich all children and teachers.
- We strengthened the explanation of the intercultural approach, its origins, and benefits through references to relevant literature.
- We cited the useful sources you suggested such as those from the Intercultural Education Journal.
- We eliminated repetitions throughout the article.
- We removed editing notes in the conclusion and carefully read the entire article to correct minor editing errors.

We hope that these revisions address your concerns and improve the overall quality of our manuscript. Thank you again for your valuable feedback.
